# Brain reward responses to food stimuli among female monozygotic twins discordant for BMI

**DOI:** 10.1007/s11682-017-9711-1

**Published:** 2017-06-08

**Authors:** Stieneke Doornweerd, Eco J. De Geus, Frederik Barkhof, Liselotte Van Bloemendaal, Dorret I. Boomsma, Jenny Van Dongen, Madeleine L. Drent, Gonneke Willemsen, Dick J. Veltman, Richard G. IJzerman

**Affiliations:** 1 Department of Internal Medicine, VU University Medical Centre, De Boelelaan 1117, 1081 HV Amsterdam, The Netherlands; 2 EMGO+ Institute for Health and Care Research, VU University Medical Centre, Amsterdam, The Netherlands; 3 Neuroscience Campus Amsterdam, VU University Medical Centre, Amsterdam, The Netherlands; 4 Biological Psychology, Vrije Universiteit, Amsterdam, The Netherlands; 5 Department of Radiology and Nuclear Medicine, VU University Medical Centre, Amsterdam, The Netherlands; 6 Department of Internal Medicine/Endocrine Section, VU University Medical Centre, Amsterdam, The Netherlands; 7 Department of Psychiatry, VU University Medical Centre, Amsterdam, The Netherlands

**Keywords:** Obesity, Reward, Food, Monozygotic, Genetic, fMRI

## Abstract

Obese individuals are characterized by altered brain reward responses to food. Despite the latest discovery of obesity-associated genes, the contribution of environmental and genetic factors to brain reward responsiveness to food remains largely unclear. Sixteen female monozygotic twin pairs with a mean BMI discordance of 3.96 ± 2.1 kg/m^2^ were selected from the Netherlands Twin Register to undergo functional MRI scanning while watching high- and low-calorie food and non-food pictures and during the anticipation and receipt of chocolate milk. In addition, appetite ratings, eating behavior and food intake were assessed using visual analog scales, validated questionnaires and an ad libitum lunch. In the overall group, visual and taste stimuli elicited significant activation in regions of interest (ROIs) implicated in reward, i.e. amygdala, insula, striatum and orbitofrontal cortex. However, when comparing leaner and heavier co-twins no statistically significant differences in ROI-activations were observed after family wise error correction. Heavier versus leaner co-twins reported higher feelings of hunger (*P* = 0.02), cravings for sweet food (*P* = 0.04), body dissatisfaction (*P* < 0.05) and a trend towards more emotional eating (*P* = 0.1), whereas caloric intake was not significantly different between groups (*P* = 0.3). Our results suggest that inherited rather than environmental factors are largely responsible for the obesity-related altered brain responsiveness to food. Future studies should elucidate the genetic variants underlying the susceptibility to reward dysfunction and obesity.

**Clinical Trial Registration Number:** NCT02025595.

**Electronic supplementary material:**

The online version of this article (doi:10.1007/s11682-017-9711-1) contains supplementary material, which is available to authorized users.

## Introduction

Increasing evidence suggests that altered brain reward responses to food stimuli promote excessive eating, making people prone to the development of obesity (Berridge et al. 2010; Volkow et al. 2011). In studies using functional magnetic resonance imaging (fMRI), we and others demonstrated that obese compared to lean individuals have higher activity in reward-related areas, such as the insula, striatum and amygdala when watching palatable food images or cues that predict palatable food receipt (Pursey et al. 2014; Stice et al. 2008b; Ten Kulve et al. 2015; van Bloemendaal et al. 2014), as well as less activation in response to the actual receipt of palatable food (Stice et al. 2008a; van Bloemendaal et al. 2015). Increased activity to food cues in obese individuals may reflect higher craving for food, while decreased activation to actual consumption may reflect a reward deficit leading to compensatory overeating (Stice and Yokum 2016).

Body weight regulation is known to be influenced by a multitude of genetic and environmental factors and their interactions (Marti et al. 2004). Results from twin and adoption studies suggest that 40–70% of inter-individual variability in BMI is explained by genetic factors whereas the shared environment of family members, such as living in the same household, has only a limited impact (Schousboe et al. 2004; Van Dongen et al. 2013). Previous neuroimaging studies observed altered brain responses to food stimuli in individuals with rare monogenic forms of hyperphagia and obesity (Farooqi et al. 2007; van der Klaauw et al. 2014), and in carriers of risk alleles of genes associated with common obesity, such as the *FTO*-gene (Heni et al. 2014; Karra et al. 2013). These findings indicate that altered reward function in the brain is a feature of the genetic predisposition to excessive eating and weight gain.

In addition to heredity, environmental factors play an important role in body weight regulation and obesity development, as evidenced by the rapid increase in obesity prevalence during a time period in which gene pools of populations remained relatively stable. Further evidence for a role for the environment comes from monozygotic twins which, despite identical genetic backgrounds, can differ in body weight and dietary intake (Doornweerd et al. 2016; Van Dongen et al. 2015). In contrast to genetic factors, the influence of environmental factors on brain reward responsiveness to food has not been investigated. Although a recent fMRI study investigated brain responsiveness to food in monozygotic twins (Melhorn et al. 2016), the focus of this study was on the degree of *similarity* within the twins, which provides a measure of genetic influences, whereas focusing on intra-pair *differences* allows for the investigation of unique environmental influences. Since monozygotic twins are genetically identical, all differences between the twins must be ascribed to unique environmental factors.

Therefore, in the present study we used a special design of monozygotic twins discordant for BMI to investigate the influence of environmental factors on individual differences in brain reward responsiveness to visual food cues and to the anticipation and receipt of a palatable food stimulus as measured with fMRI.

## Methods

### Subjects

The selection of participants from the Netherlands Twin Registry (Willemsen et al. 2013) was done as described in detail previously (Doornweerd et al. 2016). In short, out of 2775 monozygotic twin pairs, 54 female monozygotic twin pairs were selected as having a BMI discordance of ≥2 kg/m^2^ based on previously measured BMI (Willemsen et al. 2010). Only females were selected because of earlier reported sex-differences in food-related brain activations and larger responses in females compared to males (Pursey et al. 2014). Twin pairs were invited by letter and contacted by telephone to check their willingness and eligibility. Inclusion criteria comprised age range 18–75 years, stable body weight (<5% reported change during the previous 3 months) and normoglycemia as defined by fasting glucose <7.0 mmol/L on the day of the test visit. Exclusion criteria were current diabetes mellitus, serious heart, liver or renal disease, malignancies, uncontrolled thyroid disease, neurological or psychiatric disease including eating disorders and depression (assessed by the Center for Epidemiologic Studies Depression Scale (Schroevers et al. 2000)), pregnancy or breast feeding, MRI contra-indications, alcohol or drug abuse and the use of glucose-lowering drugs or psychoactive medication. Fourteen twin pairs were unwilling to participate mostly because of reported lack of time. Twenty-one twin pairs were excluded due to exclusion criteria as published previously (Doornweerd et al. 2016).

Thus, 16 female monozygotic twin pairs were willing and eligible to participate. Zygosity assessments were based on DNA genotyping performed on Affymetrix 6.0 (Willemsen et al. 2013). One pair was part of a monozygotic triplet. The study protocol was approved by the medical ethics committee of the VU University Medical Center and performed in accordance with the Helsinki Declaration. All subjects provided written informed consent.

### Measures

#### Clinical assessments

Participants were asked to consume their regular meals the day prior to the test visit, but to refrain from eating or drinking for 12 h and heavy exercise for 24 h preceding their test visit. Both co-twins of a pair arrived at the research clinic between 8 and 10 AM on the same day. Information on socio-demographics and health was collected using oral interviews. Anthropometric data were measured in a standardized manner as described previously (Doornweerd et al. 2016).

#### Questionnaires

Before the scanning session participants were asked to rate their feelings of appetite on a Likert scale ranging from 0 (‘not at all’ or ‘nothing at all’) to 10 (‘extremely’ or ‘a lot’) (Hill et al. 1995; van Bloemendaal et al. 2014). Participants were asked the questions 1) How hungry are you now? 2) How full are you now? 3) How much could you eat right now? 4) How much is your desire right now to eat something sweet/savoury/fat? The Dutch Eating Behavior Questionnaire (DEBQ) (Van Strien et al. 1986), a 33-item validated tool to assess eating behavior, was used to assess emotional, external and restrained eating. The Eating Disorder Inventory (EDI) version 2 (Garner and Olmsted 1986) was used to assess 3 psychological aspects relevant for eating disorders (i.e. drive for thinness, bulimia and body dissatisfaction). In these analyses we used the untransformed scoring system, with ratings ranging from one to six (Schoemaker et al. 1994).

#### Food stimuli ratings

After the scanning session participants viewed all food pictures that were presented during the fMRI session and rated each picture on how attractive the food in the picture appeared to them at that moment on a Likert scale ranging from 1 (‘not at all’) to 7 (‘extremely’). On a similar scale participants rated the attractiveness of the taste of chocolate milk and tasteless solution used in the fMRI experiment.

#### Ad libitum lunch meal

At the end of the test visit participants were presented a standardized varied choice meal (van Bloemendaal et al. 2014; van Bloemendaal et al. 2015). The meal consisted of white and multigrain bread, a mixed green salad, orange juice, Dutch cheese, fresh meats, margarine, mayonnaise, peanut butter, jam, cake, a chocolate muffin, a banana and an apple. Twins were seated at two separate tables, each on the other side of the room. They could eat as much as they wanted and were not informed that their consumption of food was being monitored. Food items were coded with the corresponding NEVO-code (Dutch Food Composition Table) (RIVM 2013). Intake of energy (kcal) and percentages of kcal derived from total fat, saturated fat, unsaturated fat, protein and carbohydrates was determined.

### Imaging paradigms

Imaging paradigms used in the current study were described in detail previously (Ten Kulve et al. 2016; Ten Kulve et al. 2015; van Bloemendaal et al. 2014; van Bloemendaal et al. 2015).

#### Food pictures

**Fig. 1:**
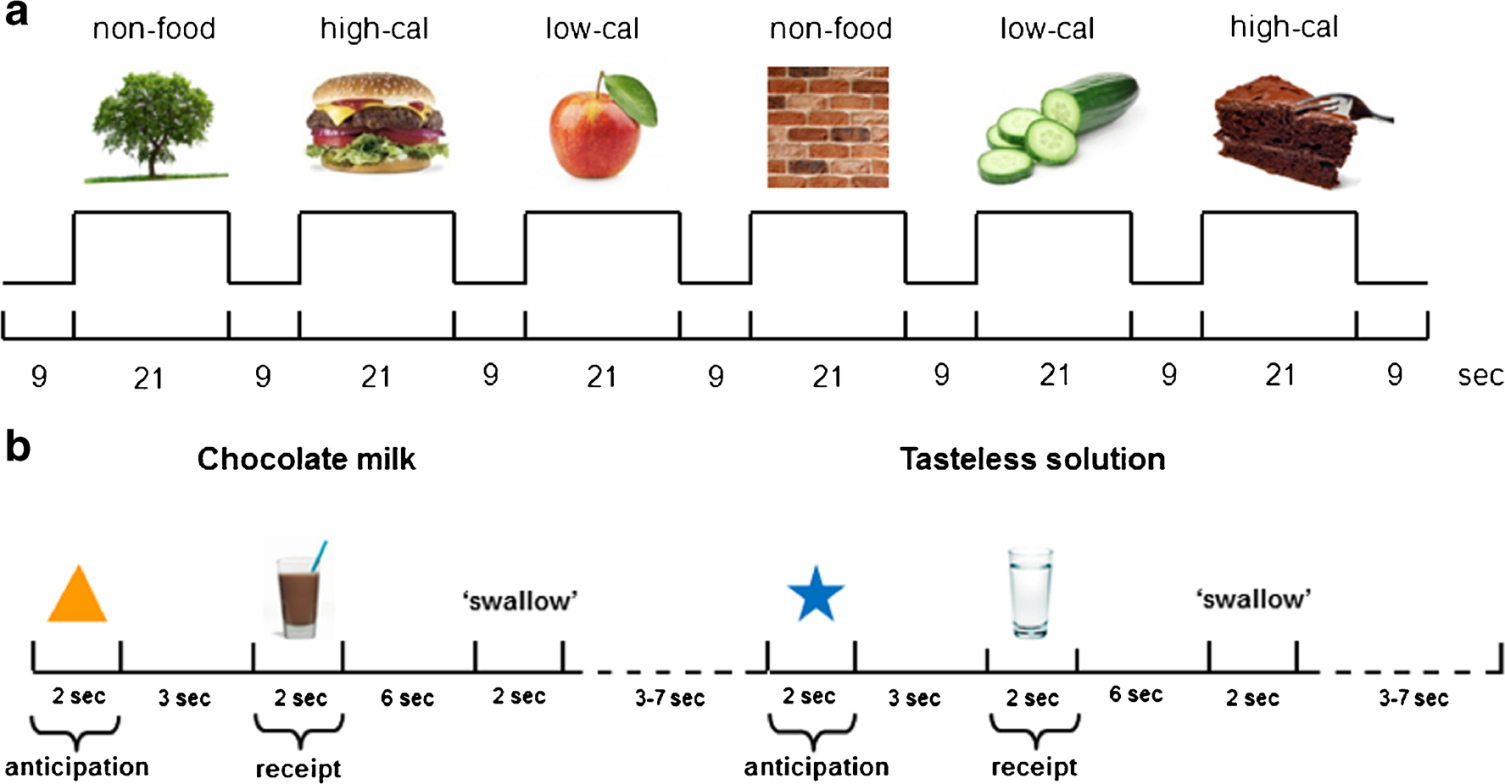
Example of timing of picture presentation during the food picture fMRI paradigm (**a**) and example of timing of cue presentation and stimuli delivery during the chocolate milk fMRI paradigm (**b**). Cal, calorie

Pictures were presented in 3 runs comprising 6 blocks each: 2 blocks of high-calorie (HC) food (e.g. chocolate cake, ice-cream, pizza, and hamburgers), 2 blocks of low-calorie (LC) food (e.g. apples, broccoli, tomatoes and green salads) and 2 blocks of non-food items (e.g. trees, flowers, rocks and bricks) (Fig. 1a). Within each block 7 pictures were presented for 2.5 s each, separated by a 0.5 s blank screen. Participants were instructed to attentively watch each picture. One hour after the scanning session a recognition test was performed. The recognition test consisted of 20 pictures of which subjects needed to identify 10 pictures that were previously shown in the scanner.

#### Palatable food stimuli

Each fMRI run included 64 trials. Chocolate milk (Chocomel; 86 kcal, 2.7 g fat, 11.8 g sugar per 100 ml) was used as a palatable food stimulus. A tasteless solution was used as a neutral stimulus, designed to mimic the natural taste of saliva (Stice et al. 2008a). During each trial an image was presented (either an orange triangle or a blue star) that signaled the delivery of either 0.4 ml chocolate milk or tasteless solution **(**Fig. 1b **)**. Images were presented for 2 s (i.e. anticipation) in random order, followed by 3 s of blank screen with a fixation cross and 2 s of stimulus delivery (i.e., receipt). Participants were instructed to keep the solution in their mouth during 6 s until the sign ‘swallow’ appeared. The next trial was started after a jitter of 3–7 s. In 40% of the events, the cue was not followed by a stimulus delivery (Stice et al. 2008b).

### Image acquisition

Imaging data were acquired using a 3.0 Tesla GE Signa HDxt scanner (General Electric, Milwaukee, WI, USA). For structural imaging, T1 weighted scans were acquired using a 3D fast spoiled gradient-echo sequence. For the functional data, a T2* weighted gradient echo-planar imaging sequence was used (repetition time/echo time = 2160/30 msec, flip angle 80°, slice thickness 3 mm, matrix size 64 × 64, 211 × 211 mm^2^ field of view, voxel size 3 × 3 × 3 mm, 40 slices).

### Data analysis

#### Clinical data

Clinical and behavioral data were analyzed using IBM SPSS Statistics (version 20, IBM Corp., 2011, Armonk, NY). Results are expressed as mean ± SD. Differences between the leaner and heavier co-twins were tested with paired sample t-tests for continuous variables (Altman 1991), McNemar tests for dichotomous variables and Wilcoxon signed-ranks tests for ordinal data.

#### Imaging data

Data were pre-processed using SPM8 software (Wellcome Trust Centre for Neuroimaging, London, UK) run within Matlab R2012a (Mathworks, Inc.). Due to obvious artifacts resulting from a metal implant in the spinal cord, the data set of one woman (and her twin sister in case of paired analyses) was excluded from further fMRI analyses. Of the remaining data, the origin of each volume was aligned to the anterior commissure. Functional images were realigned to the first volume and slice-time corrected to the onset of the middle slice. After co-registration to T1 scans, volumes were normalized into standard Montreal Neurological institute (MNI) space. Volumes were resliced into 3 × 3 × 3 mm voxels and spatially smoothed using a 8 mm full width at half maximum Gaussian kernel. The functional data were passed through a high-pass filter (cutoff 128 s). No data set showed within-run head movement of >2.5 mm in translation or >2.5° in rotation.

Block-design BOLD-responses were analyzed within the context of the general linear model. At the first level, for each participant contrast images were generated for 1) watching food vs. non-food pictures, 2) watching high-calorie vs. non-food pictures, 3) anticipating chocolate milk vs. baseline, and 4) chocolate milk receipt vs. baseline. Baseline was defined as the jittered time between trials, excluding the first 3 s. To specifically assess the effect of anticipating and receiving a palatable taste stimulus as opposed to anticipating and receiving a taste stimulus in general, contrasts were also generated for 5) anticipation of chocolate milk vs. tasteless solution and 6) receipt of chocolate milk vs. tasteless solution.

Based on previous studies on food reward and motivation (Pursey et al. 2014; Stice et al. 2008b) we selected the amygdala, insula, caudate nucleus, putamen and orbitofrontal cortex (OFC) as our a priori regions of interest (ROIs). We defined functional ROIs specific to our tasks and contrasts based on the orthogonal main effects of all participants in this study (Friston et al. 2006; Kriegeskorte et al. 2009). To this end, contrasts of all participants were entered in a one-sample t-test and, for each contrast, a statistical map was calculated. An implicit anatomical mask containing our bilateral ROIs (created with the Wake Forest University (WFU) toolbox, Winston-Salem, NC, USA) was used to visualize brain activation in our a priori anatomical ROIs only. Statistical maps of the one-sample t-tests were thresholded at *P* < 0.001 uncorrected. Montreal Neurological Institute (MNI) coordinates of significantly activated peak voxels were used to create contrast-specific ROIs, by using spheres around the peaks with a radius of 10 mm (or 5 mm for amygdala). Group differences in contrast-specific ROI activations between leaner and heavier co-twins were examined with paired t-test in SPM using a threshold of *P* < 0.05 family wise error (FWE) corrected for small volume. In addition to ROI-analyses, results are reported of regions not of our a priori interest when *P* < 0.05 FWE whole brain corrected.

## Results

### Clinical characteristics

We included 16 female monozygotic twin pairs with a mean BMI difference of 3.96 ± 2.1 kg/m^2^ (range 0.7–8.2) and a mean age of 48.8 ± 9.8 (Table 1). After excluding the twin pair comprising the participant with imaging artefacts, the mean BMI discordance was 4.2 ± 1.9 kg/m^2^ (range 1.0–8.2). Without exception, metabolic risk factors were less favorable in the heavier than in the leaner co-twins, although only lower HDL-cholesterol and higher HDL/total cholesterol ratio in the heavier co-twins were statically significant. All subjects had normal fasting glucose levels.

**Table 1:** Characteristics of leaner and heavier co-twins

	Leaner co-twin (*n* = 16)	Heavier co-twin (*n* = 16)	*P*-value
Age (y)	49.8 ± 9.8	49.8 ± 9.8	-
Weight (kg)	68.9 ± 9.2	80.5 ± 11.0	< 0.001
BMI (kg/m^2^)	24.4 ± 3.1	28.4 ± 3.5	< 0.001
Waist-to-hip ratio	0.80 ± 0.1	0.84 ± 0.1	< 0.05
Percentage body fat (%)	32.0 ± 6.1	37.8 ± 6.1	< 0.001
Fasting glucose (mmol/L)	4.7 ± 0.3	4.8 ± 0.3	0.5
HbA1c (mmol/mol)	36.3 ± 2.6	36.7 ± 2.6	0.3
Total cholesterol (mmol/L)	5.2 ± 1.1	5.3 ± 1.2	0.8
HDL cholesterol (mmol/L)	2.0 ± 0.4	1.7 ± 0.4	0.05
LDL cholesterol (mmol/L)	2.9 ± 1.0	3.2 ± 1.2	0.3
Ratio total / HDL cholesterol	2.7 ± 0.6	3.2 ± 1.0	0.01
Triglycerides (mmol/L)	0.8 ± 0.2	0.9 ± 0.3	0.1

Mean ± SD, all biochemical assessments are done in the fasted state

*HbA1c* glycated hemoglobin, *HDL* high-density lipoprotein, *LDL* low-density lipoprotein

Subjects used the following medication: thyroid hormone replacement medicines (*n* = 5, in 3 twin pairs), oral contraceptives (*n* = 4, in 3 twin pairs), antihypertensive medication (*n* = 9, in 7 twin pairs) and statins (*n* = 8, in 5 twin pairs). Leaner and heavier co-twins were comparable for self-reported daily smoking (*P* = 0.5), handedness (*P* = 1.0) and menopausal status (*P* = 0.7). Of the included women, 6 were daily smokers: in 2 pairs both co-twins smoked and in 2 pairs only the leaner co-twin smoked. Two women were left handed: 1 leaner and 1 heavier co-twin in different pairs. Thirteen women were premenopausal (defined as having a regular menstrual cycle): 7 leaner and 6 heavier co-twins in 7 pairs. In premenopausal women we initially aimed to perform all scans during the follicular phase (Dreher et al. 2007) defined as on day 1–12 counting forward from the start of the menstruation. However, since both co-twins of a pair were scanned on the same day, this was not always feasible. Nevertheless, no significant group differences were present in menstrual cycle phase, with 3 women scanned during the follicular phase in each group (*P* = 0.3).

### Behavioral measures

#### Questionnaires

**Fig. 2:**
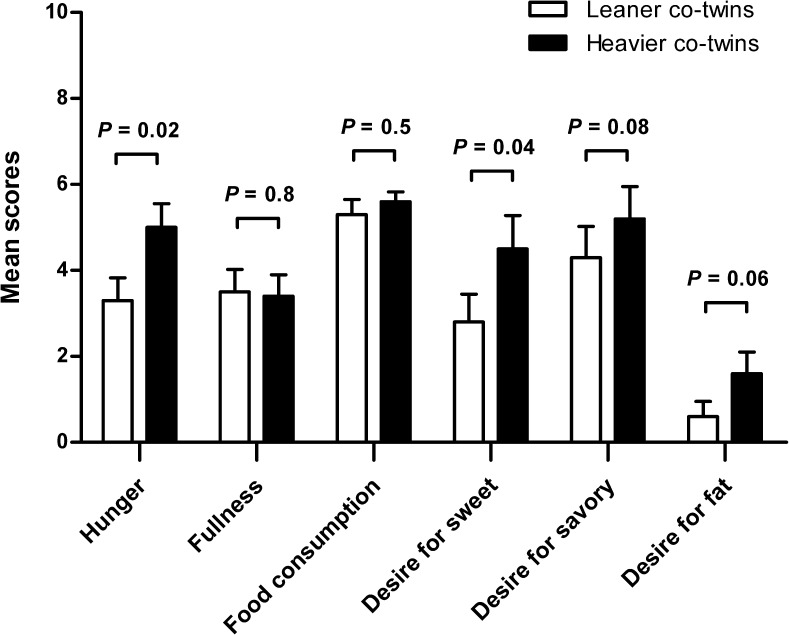
Mean ± SEM hunger and appetite ratings of leaner and heavier co-twins prior to the scanning session on a scale from 1 to 10 for the questions 1) How hungry are you? 2) How full are you? 3) How much food could you eat right now? 4) How strong is your desire right now to eat something sweet / savory / fat?

**Fig. 3:**
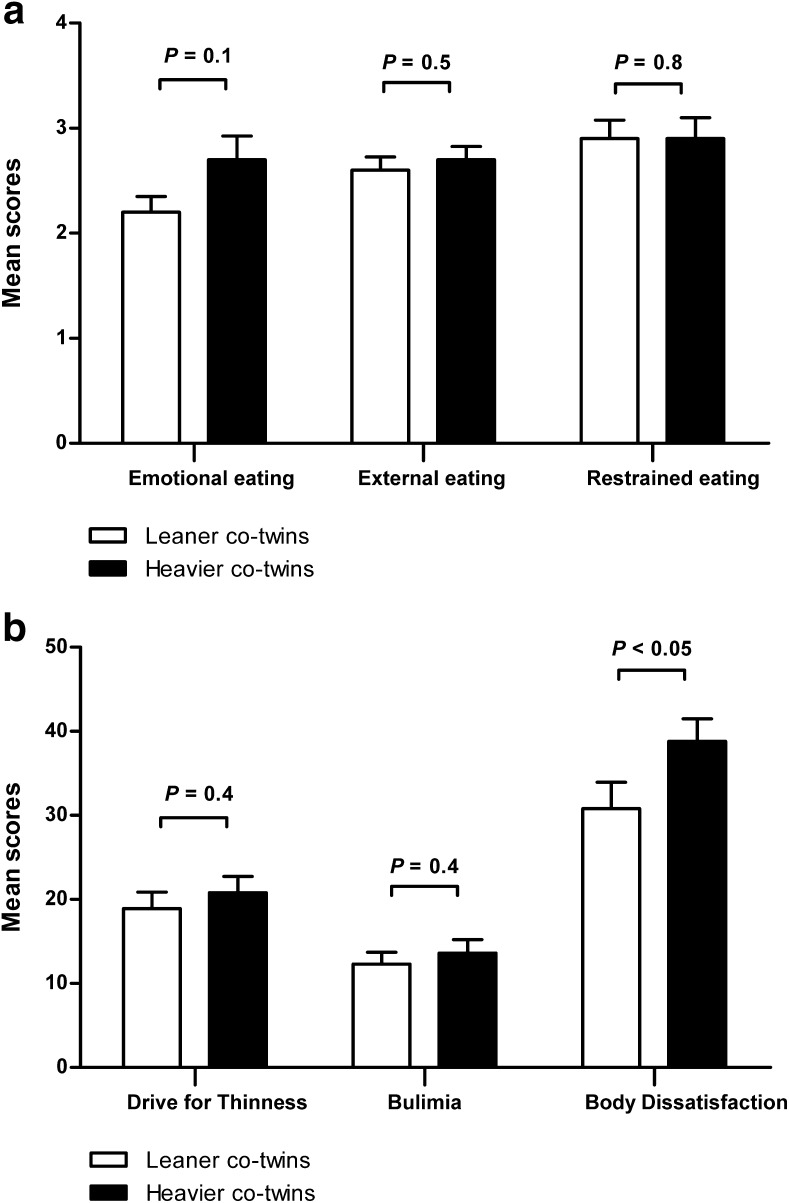
Mean scores ± SEM of leaner and heavier co-twins on (**a**) emotional, external and restraint eating, and (**b**) drive for thinness, bulimia and body dissatisfaction

Heavier co-twins reported higher feelings of hunger (*P* = 0.02) and desire to eat something sweet (*P* = 0.04) as compared to the leaner co-twins prior to the scanning session (Fig. 2), while there was a trend in desire to eat something savory (*P* = 0.08) and something high in fat (*P* = 0.06). Heavier co-twins tended to score higher on emotional eating (*P* = 0.1 Fig. 3a), and significantly scored higher on body dissatisfaction (*P* < 0.05 Fig. 3b) than leaner co-twins.

#### Food stimuli ratings

Participants in both groups rated the low-calorie food pictures as more appealing than the high-calorie food pictures (5.3 ± 0.9 vs. 3.8 ± 0.6; *P* < 0.001 in leaner co-twins; and 4.8 ± 0.8 vs. 4.0 ± 1.0; *P* < 0.05 in heavier co-twins). Both groups rated the chocolate milk and tasteless solution as equally appealing (4.9 ± 1.2 vs. 5.1 ± 1.4; *P* = 0.7 in leaner co-twins; 5.3 ± 1.8 vs. 4.9 ± 1.3; *P* = 0.3 in heavier co-twins). Furthermore, leaner and heavier co-twins performed similarly on the image recognition test after the scan (*P* = 0.9), with mean percentages of images correctly recognized of 83.8 ± 8.7 vs. 84.1 ± 12.3, respectively.

#### Ad libitum lunch meal

Total energy intake during the lunch meal following the scanning session was not significantly different between heavier and leaner co-twins, (815 ± 212 vs. 763 ± 188 kcal respectively, *P* = 0.3). There were also no differences in macronutrients intake between the groups (data not shown).

### Brain responses to food pictures

**Fig. 4:**
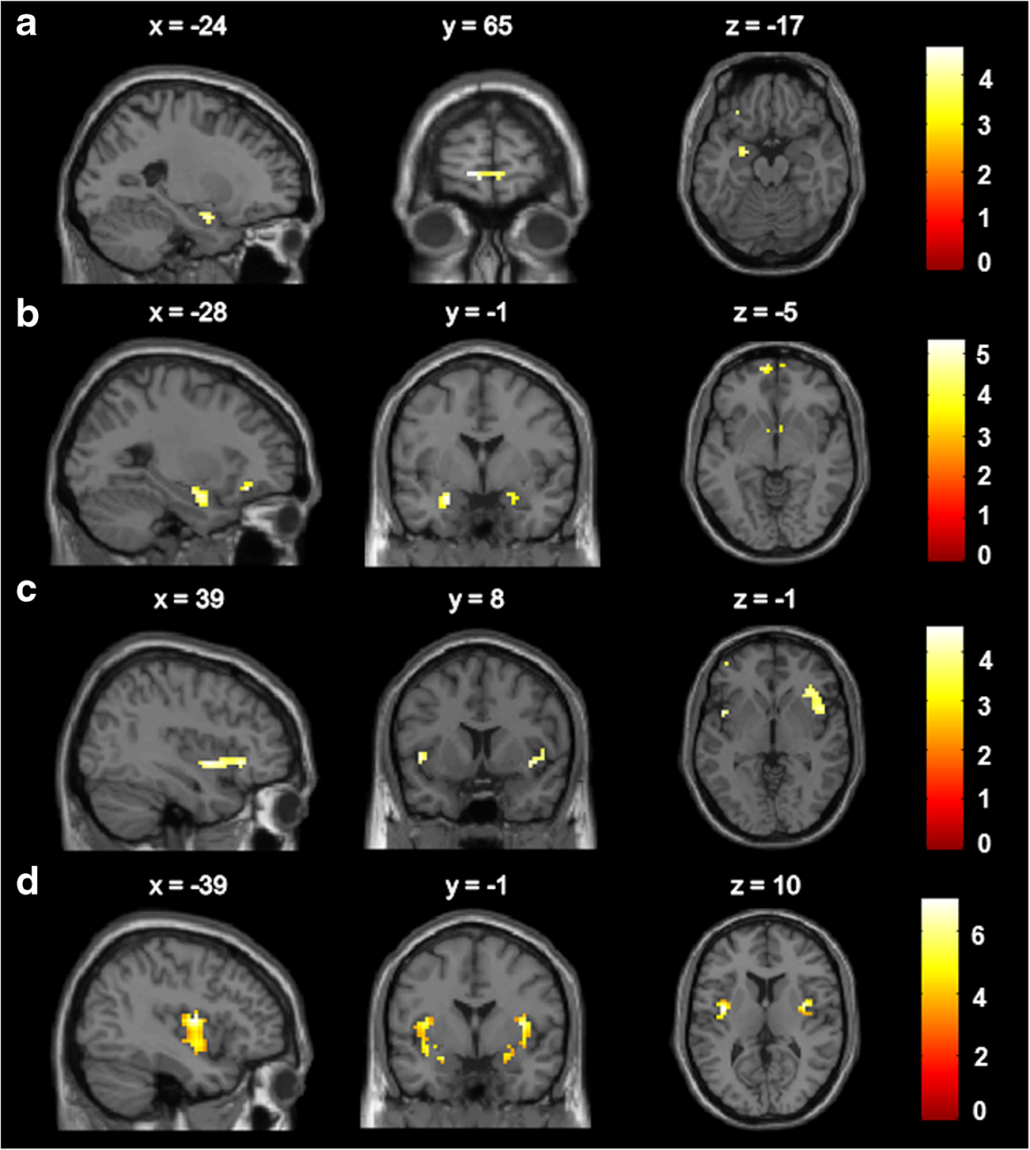
Main activations in a priori anatomical ROIs in the total group of participants with threshold *P* < 0.001 uncorrected for the contrasts (**a**) watching food vs. non-food pictures, (**b**) watching high-calorie vs. non-food pictures, (**c**) anticipation of chocolate milk vs. baseline, and (**d**) receipt chocolate milk vs. baseline. *Colour bar* represents T-value

In the overall group of women, we observed a significant main effect of watching food vs. non-food pictures within our a priori ROIs, in particular the left amygdala and bilateral orbitofrontal cortex (OFC) (Table 2 and Fig. 4). Watching high-calorie vs. non-food pictures resulted in activation of bilateral amygdala, bilateral OFC, bilateral caudate nucleus and left insula. Main effects of tasks in other regions of the brain (*P* < 0.05 FWE whole brain corrected) are presented in **Supplementary** Table 1.

**Table 2:** Main effects of tasks in ROIs

	Side			MNI			
		k	T	x	y	z	*P*-value
Food vs. non-food pictures							
OFC	L	24	4.6	−12	65	−2	3.9 × 10^−5^
	R		3.9	6	65	−2	2.7 × 10^−4^
	L	1	3.7	−30	32	−17	4.1 × 10^−4^
Amygdala	L	15	4.2	−24	−1	−20	1.0 × 10^−4^
High-calorie vs. non-food pictures							
Amygdala	L	36	5.3	−24	−1	−20	4.8 × 10^−6^
	R	8	4.1	21	−4	−17	1.5 × 10^−4^
OFC	L	13	5.0	−27	32	−14	1.3 × 10^−5^
		24	4.9	−12	65	−2	1.7 × 10^−5^
	R		3.8	3	65	−2	3.5 × 10^−4^
Insula	L	3	4.1	−36	5	−14	1.3 × 10^−4^
		7	4.0	−36	−7	4	1.9 × 10^−4^
Caudate nucleus	L	4	3.9	−9	14	−2	2.9 × 10^−4^
		1	3.5	−6	5	−5	7.1 × 10^−4^
	R	2	3.7	6	5	−5	3.9 × 10^−4^
Anticipation chocolate milk vs. baseline							
Insula	R	111	4.5	39	2	−5	4.8 × 10^−5^
OFC	R		4.4	36	26	−8	7.1 × 10^−5^
Insula	R		4.3	42	11	−5	8.2 × 10^−5^
		2	3.4	36	20	13	9.1 × 10^−4^
	L	7	4.3	−45	8	−2	7.9 × 10^−5^
		1	3.5	−36	−10	−8	8.0 × 10^−4^
		1	3.4	−27	26	−5	9.0 × 10^−4^
OFC	L	1	3.5	−42	53	−2	8.1 × 10^−4^
Receipt chocolate milk vs. baseline							
Insula	L	96	7.0	−39	−4	10	4.2 × 10^−8^
	L		5.5	-36	−4	−8	2.7 × 10^−6^
	R	56	6.2	39	−1	10	4.1 × 10^−7^
	R		4.6	39	−1	−2	3.9 × 10^−5^
Amygdala	L	15	5.7	−24	−1	−17	1.6 × 10^−6^
	R	16	4.5	27	−1	−14	5.2 × 10^−5^

Montreal Neurological Institute (MNI) coordinates of peak voxels activated in a priori anatomical ROIs in the total group of participants with threshold *P* < 0.001 uncorrected. Reported *P*-values are uncorrected

*K* cluster size, *T* T-statistic, *OFC* orbitofrontal cortex, *L* left, *R* right

When comparing groups for mean activation in the contrast-specific ROI’s, no significant differences were observed between leaner and heavier co-twins in watching food vs. non-food pictures or high-calorie vs. non-food pictures (FWE corrected for small volume). Post hoc exploration at a more lenient threshold of *P* < 0.001 in a priori anatomical ROIs also revealed no significant differences between leaner and heavier co-twins. Additional analyses were performed using anatomical ROIs based on the ALL atlas included in the WFU Pickatlas toolbox. Again no significant differences in ROI activation between leaner and heavier co-twins were found.

### Brain responses to anticipation and receipt of palatable food

In the overall group of women, we observed a significant main effect of chocolate milk anticipation vs. baseline in bilateral insula and bilateral OFC (Table 2 and Fig. 4). The receipt of chocolate milk vs. baseline significantly activated bilateral insula and bilateral amygdala. Main effects of task in other regions of the brain (*P* < 0.05 FWE whole brain corrected) are presented in Supplementary Table 1. When contrasted to the tasteless solution, no main effects of chocolate milk anticipation or receipt were observed in our ROIs.

When comparing groups for mean activation in the contrast-specific ROI’s, no significant differences were observed between leaner and heavier co-twins for the anticipation of chocolate milk vs. baseline or the receipt of chocolate milk vs. baseline (FWE corrected for small volume). Post hoc exploration of group differences at a more lenient threshold of *P* < 0.001 in a priori anatomical ROIs revealed that heavier vs. leaner co-twins had lower activation during anticipation of chocolate milk vs. baseline in the left OFC (MNI -27 35–11, *T* = 3.8 cluster size k = 2 *P* = 0.0009). In contrast, heavier vs. leaner co-twins had higher activation to the receipt of chocolate milk vs. baseline in the left insula (MNI -36 11 7, *T* = 4.2 k = 4 *P* = 0.0004). Additional analyses using anatomical ROIs based on the ALL atlas included in the WFU Pickatlas toolbox did not reveal significant differences in ROI activation between leaner and heavier co-twins.

## Discussion

We used a unique design of monozygotic twins discordant for BMI to examine the influence of unique environmental factors on obesity-related alterations in brain reward responses to food. In the overall group of females we observed significant main effects of our fMRI experiments, i.e. watching (high-calorie) food pictures and the anticipation and receipt of a palatable food stimulus, in brain regions implicated in reward and motivation, such as the insula, amygdala, caudate nucleus and orbitofrontal cortex (OFC). However, when comparing heavier and leaner co-twins in activation of these regions of interest (ROIs), we observed no statistically significant differences between the groups.

These findings are of interest since in previous studies in unrelated individuals we (Ten Kulve et al. 2016; Ten Kulve et al. 2015; van Bloemendaal et al. 2014; van Bloemendaal et al. 2015) and others (Pursey et al. 2014; Rothemund et al. 2007; Stice et al. 2008b; Stoeckel et al. 2008) observed that obese relative to lean individuals have increased reward region responsiveness to palatable food images or cues that predict palatable food receipt, and decreased striatal activation to the consumption of a palatable food. The lack of these associations in our monozygotic twin design suggests that the previously observed associations between brain reward responses and obesity in unrelated individuals can be explained by genetic factors. This aligns with findings of two previous studies in twins. First, evidence for a substantial genetic influence on food reward was provided by a classical twin study showing that 75% of variability in food cue responsiveness, as examined with validated questionnaires, was explained by genetic factors (Carnell et al. 2008). More recently, a study in monozygotic twins reported greater similarity *within* twin pairs than *between* twin pairs in brain responses to visual food cues as measured with fMRI (Melhorn et al. 2016), indicating an important role of inherited factors in the brain’s appetite regulation.

These findings align with the emerging evidence that genetic variants associated with obesity are involved in the regulation of reward and appetite by the central nervous system (van der Klaauw and Farooqi 2015). Recently identified obesity-related loci have shown high expression not only in the hypothalamus, which is a key site for the central regulation of appetite, but also in the limbic system, which regulates reward, learning and motivation (Locke et al. 2015). Furthermore, neuroimaging studies have demonstrated altered brain reward responses to food stimuli in patients with monogenic forms of obesity and in individuals with common risk alleles of obesity-associated genes (such as *FTO* and *MC4R*) (Heni et al. 2014; Karra et al. 2013; van der Klaauw et al. 2014). Although many other obesity-loci are suggested to act through the brain (Locke et al. 2015), their underlying mechanism are yet to be investigated, for instance through the promising large scale collaborations on genetic variants and brain function (Medland et al. 2014).

An additional finding of our study was that actual food intake was similar in leaner and heavier co-twins during the ad libitum lunch meal, which echoes the results of the previous fMRI study in monozygotic twins in which a similar test meal was used (Melhorn et al. 2016). The results suggest that consistent inherited influences impact on actual food intake when eating to satiety, possibly mediated by reward responsiveness in the brain. However, since subjects in our study had lunch simultaneously and in the presence of the research physician, we cannot exclude the possibility that the co-twins influenced each other’s eating behavior or that bias resulted due to social desirability.

Our study is novel in that it investigates the influence of unique environmental factors on obesity-related brain reward responsiveness to food in a unique design of monozygotic twins discordant for BMI, thereby allowing for the control of genetic influences. However, there were some limitations that should be noted. First, we had only a moderate sample size because it is difficult to find discordance for BMI in MZ twins, and nearly impossible to find pairs that are extremely discordant, i.e. one being obese and the other of normal weight. The low sample size and relatively modest difference in BMI may have resulted in a power being too low to detect significant differences between leaner and heavier co-twins, particularly in our behavioral measures, such as the items on the questionnaires and food intake during the choice lunch meal. However, in previous studies from our group (Ten Kulve et al. 2016; Ten Kulve et al. 2015; van Bloemendaal et al. 2014; van Bloemendaal et al. 2015) using identical techniques and similar sample sizes, we were able to detect significant differences between lean and obese unrelated individuals, similar to other investigations (Pursey et al. 2014; Rothemund et al. 2007; Stice et al. 2008a; Stice et al. 2008b; Stoeckel et al. 2008). Compared to these previous studies, our unique design of rare monozygotic twins highly discordant for BMI but ultimately matched for confounding factors such as age, sex, shared environmental factors and genetic background should have enhanced the power of our study for investigating unique environmental influences on brain reward responses to food. With the caution that the absence of group differences within the classical inference framework does prove equivalence between groups, we tentatively interpret the absence of group differences as support for a substantial role of genetic factors on food reward regulation by the brain.

Inherent to the absence of significant differences in food reward responsiveness, the question arises which factors do explain the BMI discordance between the monozygotic twins which we investigated. First, the important role of the homeostatic regulation of feeding mediated by the hypothalamus and brainstem should not be disregarded. Since visualization of the hypothalamus is, however, hampered by its location in the brain (De Silva et al. 2012), we did not include the hypothalamus as ROI in our analyses. Secondly, the differences in BMI within pairs may have resulted from observed differences in eating behaviors, such as emotional eating in our current study, and disinhibition and restraint in previous studies (Hakala et al. 1999; Keski-Rahkonen et al. 2007). Further, differences in body weight may be ascribed to differences in physical activity. As previously published, we (Doornweerd et al. 2016) and others (Pietilainen et al. 2008; Pietilainen et al. 2010) observed significantly lower physical activity, in particular moderate-to-vigorous intensity activity in heavier compared to leaner co-twins of monozygotic twin pairs. Together, these findings suggest that the influence of unique environmental factors on body weight may be mainly through differences in homeostatic feeding pathways and deviant behaviors in eating behavior styles and physical activity, rather than through an altered reward function in the brain.

Together, our findings suggest that heritable traits have a substantial influence on reward region responses to palatable food stimuli, since no differences were observed within monozygotic twin pairs that are highly discordant for BMI. This implies that individuals that are genetically determined to increased reward responsiveness to food cues are at considerable greater risk in an environment in which the availability of energy dense palatable foods is abundant. Future studies are needed to identify the genetic variants underlying altered food reward observed in obese individuals, which may provide new clues in the development of treatment options against obesity.

## Electronic supplementary material

ESM 1 (DOCX 19 kb)
