# Supplementary material for: Brain reward responses to food stimuli among female monozygotic twins discordant for BMI
Source: Brain Imaging Behav. 2017 Jun 8;12(3):718–27. doi: 10.1007/s11682-017-9711-1 (PMC5990553; doi:10.1007/s11682-017-9711-1)
Supplement: Supplementary file 1 — (DOCX 19 kb) [file 11682_2017_9711_MOESM1_ESM.docx]

**Supplementary Table 1 Main effects of tasks whole brain**

|  |  | **Side** |  | | **MNI** | | |  |
| --- | --- | --- | --- | --- | --- | --- | --- | --- |
|  |  |  | **k** | **T** | **x** | **y** | **z** | ***P*-value** |
| **Food vs. non-food pictures** | |  |  |  |  |  |  |  |
|  | Inferior and middle occipital gyrus | L | 466 | 13.6 | -39 | -73 | -11 | 5.1 x 10^-10^ |
|  | Inferior temporal, inferior occipital and fusiform gyrus | R | 289 | 9.6 | 51 | -64 | -11 | 2.8 x 10^-6^ |
|  | Precuneus | L | 30 | 7.5 | -27 | -61 | 52 | 4.9 x 10^-4^ |
|  | Precuneus | R | 36 | 7.5 | 27 | -70 | 34 | 5.1 x 10^-4^ |
|  | Middle occipital gyrus | R | 35 | 6.5 | 36 | -82 | 7 | 0.004 |
|  | Posterior cingulate | L | 8 | 6.0 | -6 | -52 | 25 | 0.015 |
|  | Superior parietal gyrus | R | 4 | 5.9 | 30 | -58 | 55 | 0.020 |
| **High-calorie vs. non-food pictures** | | |  |  |  |  |  |  |
|  | Inferior and middle occipital gyrus | L | 658 | 11.5 | -39 | -73 | -11 | 3.6 x 10^-8^ |
|  | Inferior temporal and middle occipital gyrus | R | 583 | 11.5 | 51 | -64 | -11 | 3.7 x 10^-8^ |
|  | Precuneus | L | 50 | 7.4 | -27 | -61 | 52 | 6.0 x 10^-4^ |
|  | Superior parietal gyrus | R | 26 | 7.1 | 30 | -58 | 55 | 0.001 |
|  | Precuneus | L | 11 | 6.4 | -6 | -52 | 19 | 0.005 |
| **Anticipation chocolate milk vs. baseline** | | | |  |  |  |  |  |
|  | Middle temporal, middle occipital and fusiform gyrus | R/L | 2323 | 16.2 | 33 | -58 | -17 | 5.1 x 10^-12^ |
|  | Inferior parietal gyrus | L | 50 | 7.5 | -30 | -55 | 43 | 5.3 x 10^-4^ |
|  | Inferior parietal gyrus | R | 29 | 7.0 | 36 | -58 | 49 | 0.002 |
|  | Precentral gyrus | R | 11 | 6.5 | 45 | -1 | 40 | 0.006 |
|  | Superior temporal gyrus | R | 16 | 6.3 | 45 | -46 | 10 | 0.010 |
|  | Lingual gyrus | L | 3 | 5.8 | -12 | -70 | 4 | 0.029 |
|  | Inferior frontal gyrus | R | 3 | 5.7 | 45 | 8 | 25 | 0.037 |
| **Receipt chocolate milk vs. baseline** | | | |  |  |  |  |  |
|  | Cerebellum | L | 123 | 9.0 | -21 | -67 | -26 | 1.2 x 10^-5^ |
|  | Supramarginal gyrus | R | 91 | 8.1 | 60 | -16 | 25 | 9.8 x 10^-5^ |
|  | Precentral gyrus | R | 48 | 7.2 | 57 | 5 | 22 | 7.0 x 10^-4^ |
|  | Insula | L | 17 | 7.0 | -39 | -4 | 10 | 0.001 |
|  | Middle temporal gyrus | L | 24 | 6.7 | -48 | -55 | 4 | 0.002 |
|  | Cerebellum | R | 27 | 6.4 | 21 | -67 | -26 | 0.004 |
|  | Insula | R | 10 | 6.2 | 39 | -1 | 10 | 0.008 |
|  | Cerebellum | R | 2 | 5.9 | 15 | -73 | -38 | 0.014 |
|  | Precentral gyrus | L | 4 | 5.9 | -60 | 5 | 22 | 0.015 |
|  | Amygdala | L | 1 | 5.7 | -24 | -1 | -17 | 0.024 |
|  | Precentral gyrus | L | 1 | 5.7 | -57 | 2 | 28 | 0.024 |
|  | Cerebellum | L | 1 | 5.6 | -15 | -76 | -38 | 0.033 |
|  | Insula | L | 1 | 5.5 | -36 | -4 | -8 | 0.035 |
|  | Rolandic operculum | R | 1 | 5.4 | 51 | 5 | 13 | 0.048 |

Montreal Neurological Institute (MNI) coordinates of peak voxels activated in the total group of participants with threshold *P*<0.05 FWE whole brain corrected. Reported *P*-values are FWE whole brain corrected. K, cluster size; T, T-statistic; L, left; R, right.
